# Importance of plaque volume and composition for the prediction of myocardial ischaemia using sequential coronary computed tomography angiography/positron emission tomography imaging

**DOI:** 10.1093/ehjci/jeac130

**Published:** 2022-09-01

**Authors:** Xu Wang, Inge J van den Hoogen, Steele C Butcher, Jurrien H Kuneman, Michiel A de Graaf, Vasileios Kamperidis, Mark Boukes, Teemu Maaniitty, Jussi Schultz, Alexander R van Rosendael, Antti Saraste, Juhani Knuuti, Jeroen J Bax

**Affiliations:** Department of Cardiology, Leiden University Medical Center, Leiden, The Netherlands; Department of Cardiology, Beijing Anzhen Hospital, Capital Medical University, Beijing Institute of Heart Lung and Blood Vessel Disease, Beijing, China; Department of Cardiology, Leiden University Medical Center, Leiden, The Netherlands; Department of Cardiology, Leiden University Medical Center, Leiden, The Netherlands; Department of Cardiology, Royal Perth Hospital, Perth, Australia; Department of Cardiology, Leiden University Medical Center, Leiden, The Netherlands; Department of Cardiology, Leiden University Medical Center, Leiden, The Netherlands; Department of Cardiology, Leiden University Medical Center, Leiden, The Netherlands; Department of Communication Science at the Amsterdam School of Communication Research, University of Amsterdam, Amsterdam, The Netherlands; Turku PET Centre, Turku University Hospital and University of Turku, Turku, Finland; Turku PET Centre, Turku University Hospital and University of Turku, Turku, Finland; Department of Cardiology, Leiden University Medical Center, Leiden, The Netherlands; Turku PET Centre, Turku University Hospital and University of Turku, Turku, Finland; Heart Center, Turku University Hospital and University of Turku, Turku, Finland; Turku PET Centre, Turku University Hospital and University of Turku, Turku, Finland; Heart Center, Turku University Hospital and University of Turku, Turku, Finland; Department of Cardiology, Leiden University Medical Center, Leiden, The Netherlands; Heart Center, Turku University Hospital and University of Turku, Turku, Finland

**Keywords:** atherosclerosis, coronary artery disease, coronary computed tomography angiography, myocardial ischaemia, myocardial perfusion imaging, positron emission tomography

## Abstract

**Aims:**

Coronary atherosclerosis with a large necrotic core has been postulated to reduce the vasodilatory capacity of vascular tissue. In the present analysis, we explored whether total plaque volume and necrotic core volume on coronary computed tomography angiography (CCTA) are independently associated with myocardial ischaemia on positron emission tomography (PET).

**Methods and results:**

From a registry of symptomatic patients with suspected coronary artery disease and clinically indicated CCTA with sequential [^15^O]H_2_O PET myocardial perfusion imaging, we quantitatively measured diameter stenosis, total and compositional plaque volumes on CCTA. Primary endpoint was myocardial ischaemia on PET, defined as an absolute stress myocardial blood flow ≤2.4 mL/g/min in ≥1 segment. Multivariable prediction models for myocardial ischaemia were consecutively created using logistic regression analysis (stenosis model: diameter stenosis ≥50%; plaque volume model: +total plaque volume; plaque composition model: +necrotic core volume). A total of 493 patients (mean age 63 ± 8 years, 54% men) underwent sequential CCTA/PET imaging. In 153 (31%) patients, myocardial ischaemia was detected on PET. Diameter stenosis ≥50% (*P* < 0.001) and necrotic core volume (*P* = 0.029) were independently associated with myocardial ischaemia, while total plaque volume showed borderline significance (*P* = 0.052). The plaque composition model (χ^2^ = 169) provided incremental value for the prediction of ischaemia when compared with the stenosis model (χ^2^ = 138, *P* < 0.001) and plaque volume model (χ^2^ = 164, *P* = 0.021).

**Conclusion:**

The volume of necrotic core on CCTA independently and incrementally predicts myocardial ischaemia on PET, beyond diameter stenosis alone.

## Graphical Abstract

**Graphical Abstract:**
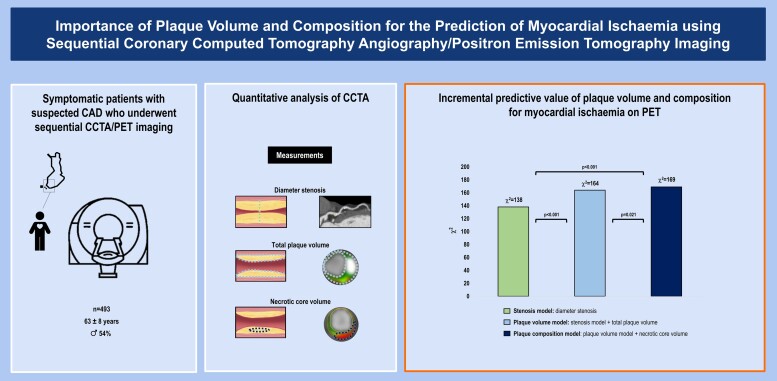
Importance of plaque volume and composition for myocardial ischaemia. Schematic representation of the study design and patients (*left panel*), the quantitative analysis of CCTA (*middle panel*), and the incremental predictive value of plaque volume and composition for myocardial ischaemia on PET (*right panel*). Images are partly derived and adjusted from Puchner *et al.*, ‘High-risk plaque detected on coronary CT angiography predicts acute coronary syndromes independent of significant stenosis in acute chest pain: results from the ROMICAT-II trial’, Journal of the American College of Cardiology, volume 64, page 687, copyright 2014, both with permission from Elsevier. CAD, coronary artery disease; CCTA, coronary computed tomography angiography; PET, positron emission tomography.

## Introduction

Myocardial ischaemia occurs when the oxygen supply relative to the demand of the myocardium is insufficient, and this can be adequately assessed using non-invasive myocardial perfusion imaging or invasive fractional flow reserve. ^1^ Although the presence of myocardial ischaemia correlates with the degree of diameter stenosis from coronary plaques, the imperfect agreement between both has been well-established. Particularly in coronary plaques with moderate stenosis (40–70% luminal narrowing) a high variability in myocardial perfusion has been described, reporting ischaemia in one-third of plaques.^2–4^ These observations have underscored the importance of identifying novel factors beyond coronary artery stenosis that may affect downstream myocardial perfusion.^1^ Recently, coronary computed tomography angiography (CCTA) has emerged as a non-invasive imaging modality that allows for the detailed volumetric measurement of different plaque components in coronary artery disease (CAD).^5,6^ Specifically, large volumes of vulnerable lipid-rich plaque—necrotic core—have been hypothesized to impair the vasodilatory response of vascular tissue, thereby inducing ischaemia.^1,7,8^ Hence, the present analysis sought to explore whether total plaque volume and necrotic core volume on CCTA are associated with myocardial ischaemia on positron emission tomography (PET), independent of diameter stenosis.

## Methods

### Study design and population

**Figure 1:**
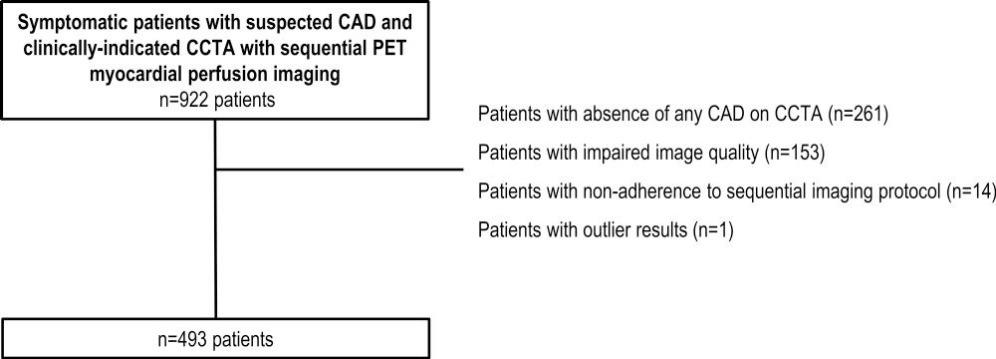
Flow chart of study population. CAD, coronary artery disease; CCTA, coronary computed tomography angiography; PET, positron emission tomography.

Consecutive symptomatic patients with suspected CAD and clinically indicated CCTA with sequential [^15^O]H_2_O PET myocardial perfusion imaging were recruited at the Turku University Hospital, Turku, Finland between 2007 and 2011. The study design has been reported earlier.^9^ In brief, a total of 922 patients were referred for CCTA, and those with a suspected obstructive stenosis on CCTA underwent subsequent PET myocardial perfusion imaging to assess the presence of myocardial ischaemia. The study protocol was approved by the ethics committee of the Hospital District of South-West Finland, and the need for written informed consent was waived. The study was performed in compliance with the Declaration of Helsinki. For the current analysis, exclusion criteria were absence of any CAD on CCTA (*n* = 261), impaired image quality (*n* = 153), non-adherence to the sequential imaging protocol (*n* = 14), and outlier results (*n* = 1). Thus, 493 patients were included (*Figure 1*).

### Sequential imaging protocol

#### Step 1: CCTA acquisition and image analysis

The CCTA was performed using a hybrid 64-detector row PET/CT scanner (GE Discovery VCT or GE D690; General Electric Medical Systems, Waukesha, WI, USA). Intravenous low-osmolar iodine (48–155 mL; 320–400 mg/mL) was given as contrast agent.^9,10^ To reach target heart rates (<60/min), intravenous metoprolol (0–30 mg) was administered before acquisition. To reach maximal coronary vasodilatation, sublingual nitroglycerin (800 μg) or isosorbide dinitrate (1.25 mg) was administered. To minimize radiation dose, prospective electrocardiogram triggering was applied whenever possible. Scans were quantitatively analysed by an independent reader (blinded to clinical or other results) in accordance with the 17-segment American Heart Association model using semi-automated software (QAngio CT Research Edition version 1.3.6; Medis Medical Imaging Systems, Leiden, the Netherlands).^5^ All coronary segments ≥1.5 mm were analysed. Tissue ≥1 mm^2^ within or adjacent to the lumen that could be distinguished from pericardial tissue, epicardial fat or lumen in >2 planes was defined as coronary plaque.^11^ Coronary plaques were evaluated for diameter stenosis, total plaque volume, and compositional plaque volume. For diameter stenosis, the maximal (quantitative) plaque-based value was selected as the patient-level value. In addition to this, cross-sectional plaque burden, minimal luminal area, lesion length, and the remodelling index were measured for these maximal stenotic plaques.^5,11,12^ For total and compositional plaque volumes, all plaque-based values were summed to provide patient-level values. Compositional plaque volume was categorized according to Hounsfield units (HU) into calcified plaque (>350 HU), fibrous plaque (131–350 HU), fibrofatty plaque (76–130 HU), and necrotic core (≤75 HU).^5,6^

#### Step 2: PET acquisition and image analysis

In patients with a suspected obstructive stenosis on initial CCTA evaluation, stress PET myocardial perfusion imaging was performed using the same PET/CT scanner.^9,10^ Intravenous [^15^O]H_2_O (Radiowater Generator, Hidex Oy, Finland) was injected as radiotracer bolus over 15 seconds.^10^ Intravenous adenosine (140 µg/kg/min) infusion was started 2 minutes prior to the stress scan to reach maximal coronary vasodilation and administered until the end of the scan. Stress scans were quantitatively analysed by an experienced reader (blinded to clinical or other results) in accordance with the 17-segment American Heart Association model using specialized software (Carimas version 1.1.0, Turku, Finland).^13,14^ Absolute stress myocardial blood flow was provided for the individual myocardial segments and total left ventricular myocardium.

### Primary endpoint

The primary endpoint was the presence of myocardial ischaemia on PET, defined as an absolute stress myocardial blood flow ≤2.4 mL/g/min in at least 1 of 17 segments.^9^ Patients without a suspected obstructive stenosis on initial CCTA evaluation did not undergo subsequent PET myocardial perfusion imaging (per study design) and were assumed non-ischaemic.

### Statistical analysis

Continuous data are shown as mean ± standard deviations or medians with interquartile ranges (IQR), dependent on distribution. Categorical data are shown as absolute numbers with percentages. Continuous data were compared with the independent sample *T* test or Mann–Whitney *U* test. Categorical data were compared with the χ^2^ test. Uni- and multivariable logistic regression analyses were performed to assess the association between selected variables and myocardial ischaemia. The following models for the prediction of myocardial ischaemia were constructed: a stenosis model (diameter stenosis ≥50%), a plaque volume model (stenosis model + total plaque volume), and a plaque composition model (plaque volume model + necrotic core volume). Particular focus was given to necrotic core volume as plaque composition, considering its link with local vasodilatory dysfunction.^1,7,8^ All models were adjusted for important clinical variables as chosen by domain expertise (X.W., I.J.v.d.H., S.C.B.). Multicollinearity between variables was ruled out as calculated with the variance inflation factor (<3 for all). Measures of association were displayed as odds ratios with 95% confidence intervals (CIs). Area under the receiver-operating characteristic curves (AUCs) of the models were compared with the DeLong’s test to evaluate discriminatory ability. χ^2^’s of the models were compared with the likelihood ratio test to evaluate goodness of model fit. Effect modification of necrotic core volume on myocardial ischaemia by total plaque volume was tested with interaction terms. A two-sided *P*-value of <0.05 indicated statistical significance, and all statistical analyses were performed with R (version 4.1.1; R Development Core Team, Vienna, Austria), SPSS (version 26; SPSS IBM Corp., Armonk, NY, USA), and STATA software (version 17; StataCorp. LLC, College Station, TX, USA).

## Results

### Study population

A total of 493 patients (mean age 63 ± 8 years, 54% men) underwent sequential CCTA/PET imaging for the assessment of CAD and myocardial ischaemia (see Supplementary data online, *Figure S1*). In 199 patients, an obstructive stenosis was excluded on CCTA, therefore they did not undergo PET myocardial perfusion imaging and were assumed non-ischaemic. In contrast, in 294 patients, an obstructive stenosis was suspected on CCTA, and in 153 (31% out of 493) patients, myocardial ischaemia was detected on subsequent PET (*Table 1*). Patients who showed myocardial ischaemia on PET were more often male (73% vs. 46%, *P* < 0.001) and more frequently had typical angina (34% vs. 23%, *P* = 0.011) compared with patients who did not show ischaemia. Moreover, cardiac risk factors including hypertension (85% vs. 75%, *P* = 0.013), dyslipidaemia (79% vs. 69%, *P* = 0.026), and diabetes mellitus (24% vs. 14%, *P* = 0.010) were more prevalent in patients with myocardial ischaemia.

**Table 1:** Baseline characteristics of study population

	Total cohort *n* = 493	Ischaemia *n* = 153	No ischaemia *n* = 340	*P*-value
Age (years)	63 ± 8	63 ± 9	63 ± 8	0.986
Male	268 (54)	112 (73)	156 (46)	<0.001
BMI (kg/m^2^)	28.0 ± 5.1	28.3 ± 4.5	27.8 ± 5.6	0.395
*Cardiac symptoms*				
Typical angina	128 (27)	52 (34)	76 (23)	0.011
Atypical angina	173 (36)	54 (36)	119 (36)	0.873
Nonanginal pain	43 (9)	10 (7)	33 (10)	0.214
Dyspnoea at exertion	136 (28)	36 (24)	100 (31)	0.124
*Cardiac risk factors*				
Hypertension	385 (78)	130 (85)	255 (75)	0.013
Dyslipidaemia	357 (72)	121 (79)	236 (69)	0.026
Diabetes mellitus	84 (17)	36 (24)	48 (14)	0.010
Family history of CAD	219 (44)	72 (47)	147 (43)	0.429
Smoking current or former	177 (36)	61 (40)	116 (34)	0.218
Number of cardiac risk factors ^a^	2.5 ± 1.1	2.7 ± 0.9	2.4 ± 1.1	<0.001
*Cardiac medication*				
Aspirin	297 (69)	111 (82)	186 (63)	<0.001
Beta–blockers	252 (58)	97 (70)	155 (53)	0.001
Calcium channel blockers	80 (19)	25 (19)	55 (19)	0.903
Renin–angiotensin system inhibitors	189 (44)	65 (46)	124 (43)	0.491
Statin	249 (58)	95 (69)	154 (53)	0.001
*Laboratory findings*				
Total cholesterol (mmol/L)	4.9 ± 1.1	4.9 ± 1.2	4.9 ± 1.0	0.861
Low-density lipoprotein (mmol/L)	2.7 ± 0.9	2.7 ± 1.0	2.7 ± 0.9	0.572
High-density lipoprotein (mmol/L)	1.5 ± 0.5	1.4 ± 0.4	1.6 ± 0.5	0.001
Triglycerides (mmol/L)	1.6 ± 1.0	1.7 ± 1.1	1.5 ± 1.0	0.085
Creatinine (µmol/L)	77.2 ± 15.4	81.2 ± 16.2	75.5 ± 14.8	<0.001

Values are presented as mean ± SD or *n* (%).

BMI, body mass index; CAD, coronary artery disease.

Including hypertension, dyslipidaemia, diabetes mellitus, family history of CAD, and smoking current or former.

### CCTA results

**Figure 2:**
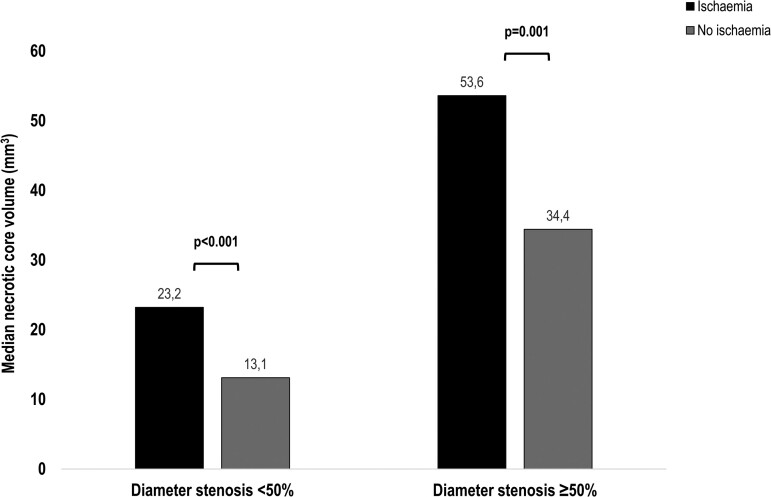
Necrotic core volumes according to obstructive status. Patients were categorized into 2 groups according to diameter stenosis: <50 and ≥50%. The black bars represent ischaemia-positive patients and the grey bars ischaemia-negative patients on PET myocardial perfusion imaging. Median necrotic core volumes (*y*-axis) are compared between groups. PET, positron emission tomography.

CCTA results of the study population are depicted in *Table 2*. Mean diameter stenosis was 39.8 ± 20.9%, and in 137 (28%) patients, a diameter stenosis ≥50% was observed on CCTA. Patients with myocardial ischaemia demonstrated more often a diameter stenosis ≥50% (60 vs. 13%, *P* < 0.001) when compared with patients without ischaemia. Furthermore, total plaque volume (371.5 mm^3^, IQR 197.8–739.4 mm^3^ vs. 127.8 mm^3^, IQR 65.0–230.8 mm^3^, *P* < 0.001) and all compositional plaque volumes (*P* < 0.001) were greater in patients with myocardial ischaemia. Also, greater necrotic core volumes were observed irrespective of their obstructive status in ischaemic patients (<50%: 23.2 mm^3^, IQR 11.4–43.7 mm^3^ vs. 13.1 mm^3^, IQR 6.3–22.5 mm^3^, *P* < 0.001; ≥50%: 53.6 mm^3^, IQR 35.1–78.4 mm^3^ vs. 34.4 mm^3^, IQR 21.1–53.9 mm^3^, *P* = 0.001; *Figure 2*).

**Table 2:** CCTA and PET results

	Total cohort *n* = 493	Ischaemia *n* = 153	No ischaemia *n* = 340	*P*-value
*CCTA*				
General				
ȃDiameter stenosis (%)	39.8 ± 20.9	55.9± 21 .3	32.6 ± 16.2	<0.001
ȃ<50%	356 (72)	61 (40)	295 (87)	<0.001
ȃ≥50%	137 (28)	92 (60)	45 (13)	<0.001
ȃTotal plaque volume (mm^3^)	169.0 (81.4–353.2)	371.5 (197.8–739.4)	127.8 (65.0–230.8)	<0.001
ȃCalcified volume (mm^3^)	28.1 (4.3–95.9)	84.9 (24.1–224.1)	14.9 (2.4–51.3)	<0.001
ȃFibrous volume (mm^3^)	85.1 (40.9–164.7)	165.5 (94.8–309.6)	62.0 (34.2–110.9)	<0.001
ȃFibrofatty volume (mm^3^)	32.0 (16.5–61.2)	63.5 (37.2–109.9)	24.1 (13.8–42.8)	<0.001
ȃNecrotic core volume (mm^3^)	20.0 (9.4–39.1)	41.3 (22.7–66.8)	14.3 (6.6–27.0)	<0.001
Maximal stenotic plaque				
ȃCross-sectional plaque burden (%)	67.5 (54.3–83.7)	84.1 (69.1–95.0)	62.4 (50.6–73.5)	<0.001
ȃMinimal luminal area (mm^2^)	3.4 (1.7–5.7)	1.4 (0.5–2.6)	4.4 (2.7–6.7)	<0.001
ȃLesion length (mm)	9.6 (5.8–14.6)	13.5 (7.4–20.5)	8.5 (5.4–12.5)	<0.001
ȃRemodelling index	0.9 ± 0.2	0.9 ± 0.2	0.9 ± 0.2	0.849
*PET myocardial perfusion imaging*				
ȃGlobal stress myocardial blood flow (mL/g/min)	–	2.3 ± 0.7 ^a^	3.8 ± 0.9 ^b^	<0.001

Values are presented as mean ± SD, median (IQR) or *n* (%).

CCTA, coronary computed tomography angiography; PET, positron emission tomography.

Within this group, regional stress myocardial blood flow for the ischaemic and remote non-ischaemic myocardium was 1.8 ± 0.3 and 3.0 ± 0.4 mL/g/min, respectively. For the ischaemic and remote non-ischaemic myocardium, this was calculated as the mean of all segments classified as ischaemic or non-ischaemic (≤2.4 and >2.4 m/g/min, respectively).

Within this group, values were only available for those who underwent subsequent PET myocardial perfusion imaging.

### PET results

PET myocardial perfusion imaging results of the study population are depicted in *Table 2*. A median of 9 segments (IQR 4–15 segments) was affected in patients with myocardial ischaemia. Patients with myocardial ischaemia demonstrated a lower global stress myocardial blood flow when compared with patients without ischaemia on subsequent PET (2.3 ± 0.7 vs. 3.8 ± 0.9 mL/g/min, *P* < 0.001). Within this group, perfusion defects fully matched the stenosis findings in 98 (64%) patients, while this was partially or not the case in 34 (22%) and 21 (14%) patients, respectively.

### Prediction of myocardial ischaemia

#### Independent predictors

Male sex, hypertension, dyslipidaemia, diabetes mellitus, number of cardiac risk factors, diameter stenosis ≥50%, total plaque volume, and all compositional plaque volumes were univariable predictors of myocardial ischaemia on PET (see Supplementary data online, *Table S1*). Different multivariable prediction models were consecutively created: a stenosis model (diameter stenosis ≥50%), a plaque volume model (stenosis model + total plaque volume) and a plaque composition model (plaque volume model + necrotic core volume; *Table 3*). In the plaque composition model, diameter stenosis ≥50% (*P* < 0.001) and necrotic core volume (*P* = 0.029) remained independently associated with myocardial ischaemia. Additionally, total plaque volume showed a borderline significant association (*P* = 0.052).

**Table 3:** Multivariable analysis for myocardial ischaemia on PET

	Stenosis model		Plaque volume model		Plaque composition model	
	OR (95% CI)^a^	*P*-value	OR (95% CI)^a^	*P*-value	OR (95% CI)^a^	*P*-value
Diameter stenosis ≥50%	10.392 (6.399–16.876)	<0.001	5.622 (3.285–9.621)	<0.001	4.909 (2.831–8.512)	<0.001
Total plaque volume (mm^3^)			1.002 (1.001–1.003)	<0.001	1.001 (1.000–1.003)	0.052
Necrotic core volume (mm^3^)					1.016 (1.002–1.031)	0.029
AUC (95% CI)	0.802 (0.758–0.845)		0.834 (0.794–0.874)	0.001^b^	0.838 (0.799–0.876)	0.260^c^

AUC, area under the receiver-operating characteristics curve; PET, positron emission tomography.

Adjusted for age, sex, and >3 cardiac risk factors including hypertension, dyslipidaemia, diabetes mellitus, family history of CAD, and smoking current or former

Compared with the stenosis model.

Compared with the plaque volume model.

#### Incremental predictive value of plaque volume and composition

**Figure 3:**
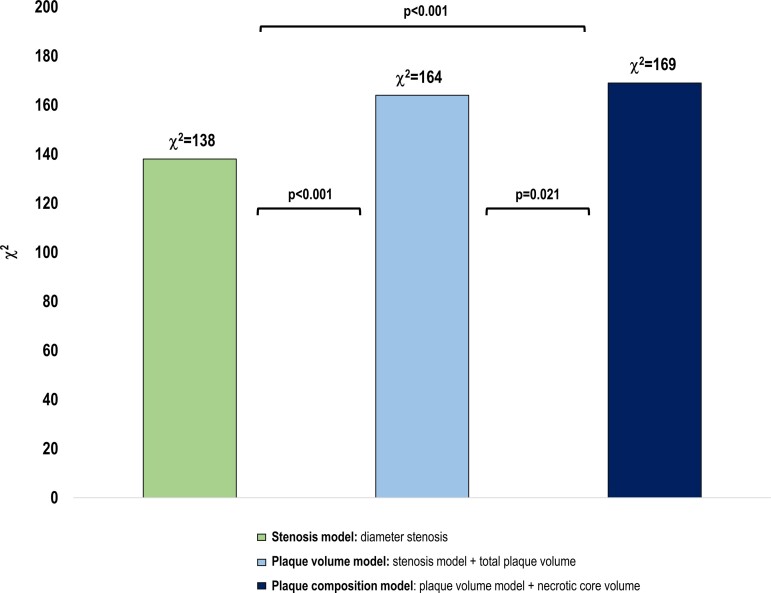
Incremental predictive value of total plaque volume and necrotic core volume. The left bar represents the stenosis model, the middle bar the plaque volume model, and the right bar the plaque composition model. χ^2^ Values (*y*-axis) are compared between the models. All models are adjusted for age, sex, and >3 cardiac risk factors including hypertension, dyslipidaemia, diabetes mellitus, family history of CAD, and smoking current or former. CAD, coronary artery disease.

A stepwise increase in AUC for discrimination of myocardial ischaemia was observed for the consecutive models: 0.802 (95% CI 0.758–0.845) for the stenosis model, 0.834 (95% CI 0.794–0.874) for the plaque volume model and 0.838 (95% CI 0.799–0.876) for the plaque composition model (*Table 3*). The plaque composition model had the highest AUC value, though this did not reach statistical significance compared with the plaque volume model (*P* > 0.05). However, the plaque composition model significantly increased the model fit, indicating that adding necrotic core volume to the model provided incremental predictive information (χ^2^ = 169 vs. χ^2^ = 164, *P* = 0.021) (*Figure 3*).

#### Interaction between plaque volume and composition

**Figure 4:**
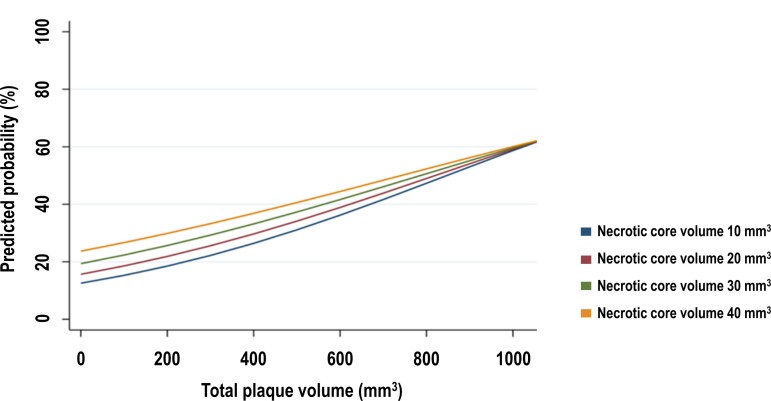
Interaction between total plaque volume and necrotic core volume. In order to show the interaction between total plaque volume and necrotic core volume, 4 values of necrotic core volume were chosen (10 mm^3^, 20 mm^3^, 30 mm^3^, 40 mm^3^). The differences between the slopes of the 4 groups reflect the interaction (*P* = 0.036). A greater necrotic core volume was associated with an increased risk of myocardial ischaemia. However, this effect was more pronounced in patients with a smaller total plaque volume and decreased with increasing total plaque volume. Predicted probabilities are calculated from the plaque composition model.

A significant interaction was demonstrated between total plaque volume and necrotic core volume, both in a crude model (*P* = 0.004) and in the adjusted (plaque composition) model (*P* = 0.036; see Supplementary data online, *Table S2*). Notably, a greater necrotic core volume was associated with an increased risk of myocardial ischaemia. This effect was more pronounced in patients with a smaller total plaque volume and decreased with increasing total plaque volume (*Figure 4*).

## Discussion

The current analysis quantitatively measured diameter stenosis, total, and compositional plaque volumes on CCTA in symptomatic patients with suspected CAD who underwent sequential CCTA/PET imaging. We investigated whether total plaque volume and necrotic core volume were associated with myocardial ischaemia on PET, independent of diameter stenosis. Our findings demonstrated that necrotic core volume on CCTA independently and incrementally predicted myocardial ischaemia on PET, beyond coronary artery stenosis alone (Graphical Abstract).^15^ These observations may explain the discrepancies that are often reported between the degree of stenosis and the presence of myocardial ischaemia.^2–4^ Of note, the effect of necrotic core volume on the presence of ischaemia was more exaggerated in patients with a smaller total plaque volume.

### Effect of stenosis on myocardial ischaemia

Prior studies have examined the effect of CCTA-derived stenosis on myocardial ischaemia using non-invasive myocardial perfusion imaging.^16–18^ For instance, Naya *et al*.^16^ evaluated 73 patients with suspected CAD who underwent CCTA and PET myocardial perfusion imaging. On a per-vessel level, a greater stenosis severity was associated with a gradual decrease in stress myocardial blood flow and myocardial flow reserve (expressed as the ratio of stress to rest myocardial blood flow). Similar results were observed on a per-patient level: a greater modified Duke CAD index, integrating both the location and severity of stenosis, translated into a reduced stress myocardial blood flow (*r* = −0.25, *P* < 0.03) and myocardial flow reserve (*r* = −0.40, *P* < 0.001). Likewise, Lin *et al*.^17^ analysed 163 patients with suspected CAD who underwent CCTA and single photon emission computed tomography. Both the segment stenosis score (*P* = 0.008) and modified Duke CAD index (*P* = 0.02) were independently associated with severe abnormalities on single photon emission computed tomography. In addition, other studies employing an invasive fractional flow reserve ≤0.80 as reference standard for myocardial ischaemia, comparably revealed stenosis severity as a strong and independent predictor of ischaemia.^19–23^

### Effect of vulnerable plaque on myocardial ischaemia

Although CCTA-derived stenosis has a well-established effect on myocardial ischaemia, discordances between the two have frequently been described.^2–4^ Consequently, measures of vulnerable plaque have been proposed to potentially affect downstream myocardial perfusion in addition to coronary artery stenosis.^1^ Shmilovich *et al*.^24^ evaluated 49 patients with a focal diameter stenosis ≥70% on CCTA who consecutively underwent single photon emission computed tomography. In patients with severely stenotic plaques, the presence of necrotic core and positive remodelling on CCTA negatively impacted myocardial perfusion, both as an absolute and relative entity (*P*≤0.004 and *P*≤0.007, respectively). Furthermore, Driessen *et al*.^22^ prospectively included 208 patients with suspected CAD who underwent CCTA and PET myocardial perfusion imaging. Next to stenosis severity, non-calcified plaque volume (integrating necrotic core volume) and the presence of positive remodelling were proved to be inversely related to stress myocardial blood flow. More specifically, a greater number of adverse plaque characteristics, including necrotic core, positive remodelling, spotty calcification, and napkin-ring sign, was associated with a stepwise decrease in stress myocardial blood flow. Various studies using an invasive reference standard for myocardial ischaemia showed similar observations.^19–23^ In agreement with our results, the majority of these studies demonstrated the independent and incremental predictive value of necrotic core volume for ischaemia.^20–23^

### Clinical implications

While not yet elucidated, underlying mechanisms have been proposed as to how a large necrotic core can influence the vasodilatory response of vascular tissue.^1,8^ First, it has been postulated that coronary plaques with lower HU on CCTA have a greater lipid-rich content, consequently inducing local oxidative stress, inflammation, and endothelial dysfunction.^25–27^ Second, histopathological studies have illustrated that coronary arteries are able to dilate in order to maintain the luminal dimensions.^28^ Coronary plaques with large necrotic cores often show positive remodelling, in which potentially the Glagovian limit is reached and no further compensation can occur that will preserve the blood flow to the myocardium.^28,29^ In the present analysis, however, no differences in remodelling index were observed between patients with and without myocardial ischaemia. To this end, it should also be mentioned that diffuse and heterogenous CAD may manifest as a continuous, graded pressure drop along the coronary arteries, thereby reducing the myocardial flow reserve and potentially contributing to downstream myocardial perfusion abnormalities.^30–33^ Yet, in the current analysis the myocardial flow reserve was not available, since in most patients only stress scans (no rest myocardial blood flow) were performed as part of the study protocol.

### Limitations

Our study had an observational design with inherent limitations, including confounding and selection bias. This was a single center registry of which only part of the patients underwent quantitative analysis of CCTA scans. Moreover, PET myocardial perfusion imaging was executed according to a sequential imaging protocol, and therefore not performed in patients without a suspected obstructive stenosis. Lastly, particular measurements, such as lesion length and calcified volume, were highly correlated with diameter stenosis or total plaque volume and could hence not be added to the multivariable analysis (because of multicollinearity).

## Conclusion

The volume of necrotic core on CCTA independently and incrementally predicts myocardial ischaemia on PET, with a more pronounced effect in patients with a smaller total plaque volume. This provides additional insight into novel factors beyond diameter stenosis that may affect downstream myocardial perfusion.

## Supplementary data

Supplementary data are available at *European Heart Journal - Cardiovascular Imaging* online.

## Supplementary Material

jeac130_Supplementary_Data

## Data Availability

Data may be available upon reasonable request to the corresponding author.
