# Supplementary material for: Importance of plaque volume and composition for the prediction of myocardial ischaemia using sequential coronary computed tomography angiography/positron emission tomography imaging
Source: Eur Heart J Cardiovasc Imaging. 2022 Sep 1;24(6):776–84. doi: 10.1093/ehjci/jeac130 (PMC10229289; doi:10.1093/ehjci/jeac130)
Supplement: jeac130_Supplementary_Data [file jeac130_supplementary_data.docx]

**SUPPLEMENTAL DATA**

**Figure S1: Sequential imaging protocol.**


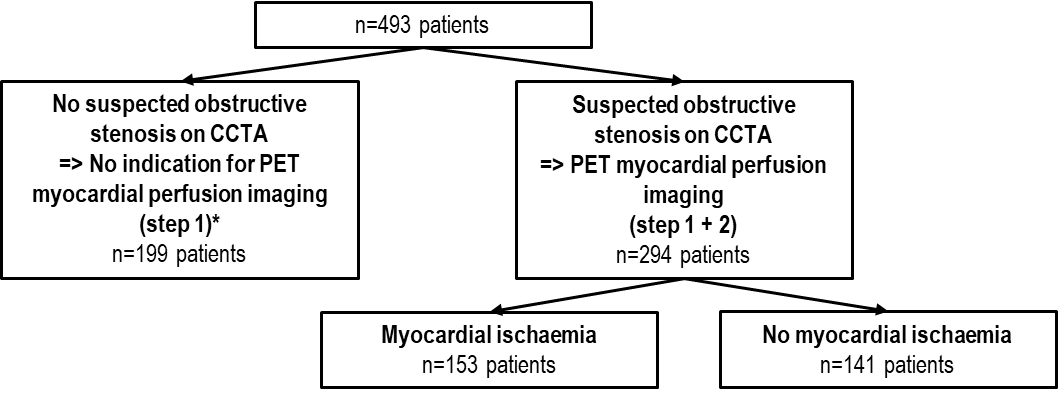
Abbreviations: CCTA, coronary computed tomography angiography; PET, positron emission tomography angiography. Definitions: *These patients did not undergo subsequent PET myocardial perfusion imaging (per study design) and were assumed non-ischaemic.

**Table S1: Univariable analysis for myocardial ischaemia on PET.**

Abbreviations: CAD, coronary artery disease; CCTA, coronary computed tomography angiography; PET, positron emission tomography. Definitions: *Including hypertension, dyslipidemia, diabetes mellitus, family history of CAD and smoking current or former.

|  | **OR (95% CI)** | **p-value** |
| --- | --- | --- |
| **Baseline characteristics** |  |  |
| Age, years | 1.000 (0.978-1.023) | 0.985 |
| Male | 3.222 (2.124-4.887) | **<0.001** |
| Hypertension | 1.884 (1.135-3.127) | **0.014** |
| Dyslipidemia | 1.666 (1.060-2.621) | **0.027** |
| Diabetes mellitus | 1.872 (1.155-3.032) | **0.011** |
| Family history of CAD | 1.167 (0.796-1.712) | 0.429 |
| Smoking current or former | 1.280 (0.864-1.898) | 0.219 |
| Number of cardiac risk factors* | 1.416 (1.175-1.707) | **<0.001** |
| **CCTA results** |  |  |
| **General** |  |  |
| Diameter stenosis ≥50% | 9.887 (6.300-15.517) | **<0.001** |
| Total plaque volume, mm^3^ | 1.003 (1.003-1.004) | **<0.001** |
| Calcified volume, mm^3^ | 1.006 (1.004-1.008) | **<0.001** |
| Fibrous volume, mm^3^ | 1.008 (1.006-1.010) | **<0.001** |
| Fibrofatty volume, mm^3^ | 1.028 91.022-1.035) | **<0.001** |
| Necrotic core volume, mm^3^ | 1.045 (1.035-1.055) | **<0.001** |
| **Maximal stenotic lesion** |  |  |
| Cross-sectional plaque burden, % | 1.067 (1.052-1.081) | **<0.001** |
| Minimal luminal area, mm^2^ | 0.600 (0.533-0.675) | **<0.001** |
| Lesion length, mm | 1.082 (1.054-1.110) | **<0.001** |
| Remodelling index | 1.116 (0.416-2.990) | **0.827** |

**Table S2: Interaction between total plaque volume and necrotic core volume.**

Definitions: *Crude model for interaction: total plaque volume, necrotic core volume and interaction term total plaque volume*necrotic core volume; ∫Adjusted model for interaction: plaque composition model (diameter stenosis ≥50%, total plaque volume, necrotic core volume, age, sex, >3 cardiac risk factors) and interaction term total plaque volume*necrotic core volume.

|  | **Crude** | **Adjusted** |
| --- | --- | --- |
|  | **interaction p-value** | **interaction p-value** |
| Total plaque volume*necrotic core volume | **0.004*** | **0.036∫** |
